# Effects of Resveratrol on the Recovery of Muscle Mass Following Disuse in the Plantaris Muscle of Aged Rats

**DOI:** 10.1371/journal.pone.0083518

**Published:** 2013-12-12

**Authors:** Brian T. Bennett, Junaith S. Mohamed, Stephen E. Alway

**Affiliations:** 1 Laboratory of Muscle Biology and Sarcopenia Division of Exercise Physiology, West Virginia University School of Medicine, Morgantown, West Virginia, United States of America; 2 West Virginia Center for Clinical and Translational Science Institute, Morgantown, West Virginia, United States of America; 3 Center for Cardiovascular, and Respiratory Sciences, West Virginia University School of Medicine, Morgantown, West Virginia, United States of America; Universidad Pablo de Olavide, Centro Andaluz de Biología del Desarrollo-CSIC, Spain

## Abstract

Aging is associated with poor skeletal muscle regenerative ability following extended periods of hospitalization and other forms of muscular disuse. Resveratrol (3,5,4’-trihydroxystilbene) is a natural phytoalexin which has been shown in skeletal muscle to improve oxidative stress levels in muscles of aged rats. As muscle disuse and reloading after disuse increases oxidative stress, we hypothesized that resveratrol supplementation would improve muscle regeneration after disuse. A total of thirty-six male Fisher 344 × Brown Norway rats (32 mo.) were treated with either a water vehicle or resveratrol via oral gavage. The animals received hindlimb suspension for 14 days. Thereafter, they were either sacrificed or allowed an additional 14 day period of cage ambulation during reloading. A total of six rats from the vehicle and the resveratrol treated groups were used for the hindlimb suspension and recovery protocols. Furthermore, two groups of 6 vehicle treated animals maintained normal ambulation throughout the experiment, and were used as control animals for the hindlimb suspension and reloading groups. The data show that resveratrol supplementation was unable to attenuate the decreases in plantaris muscle wet weight during hindlimb suspension but it improved muscle mass during reloading after hindlimb suspension. Although resveratrol did not prevent fiber atrophy during the period of disuse, it increased the fiber cross sectional area of type IIA and IIB fibers in response to reloading after hindlimb suspension. There was a modest enhancement of myogenic precursor cell proliferation in resveratrol-treated muscles after reloading, but this failed to reach statistical significance. The resveratrol-associated improvement in type II fiber size and muscle mass recovery after disuse may have been due to decreases in the abundance of pro-apoptotic proteins Bax, cleaved caspase 3 and cleaved caspase 9 in reloaded muscles. Resveratrol appears to have modest therapeutic benefits for improving muscle mass after disuse in aging.

## Introduction

Skeletal muscle comprises 40-50% of the total mass in an average young adult human and is crucial for locomotion, metabolism, generating heat, and the maintenance of posture [1,2]. However, aging induces an inevitable loss in muscle mass and function, due to a multitude of factors that together is referred to as sarcopenia [3–5]. It is thought that as much as 50% of the population over the age of 80 suffers from sarcopenia [6]. Sarcopenia has been shown to be predictive of falls [7], possibly leading to extended hospital stays and periods of immobilization. This is noteworthy, as aging muscles display an impaired or failed recovery following immobilization-induced muscle atrophy [8–10] . 

Several potential treatments have been investigated as potential countermeasure for unloading-induced atrophy, but most have only been partially successful. For example, taurine has been shown to prevent the typical slow to fast myosin shift with unloading [11] but it does not prevent muscle loss. Insulin growth factor-I overexpression during hindlimb unloading induced some potential molecular signaling improvements, especially the atrogen MurF1, but there was no overall reduction in muscle protein loss or slow to fast fiber transition loss with this treatment [12]. In addition, β-Hydroxy-β-methylbutyrate (HMB) a leucine metabolite, also provides a modest protection against skeletal muscle loss during hindlimb unloading in old rats [13], but again it does not prevent muscle wasting. Resveratrol (3,5,4’-trihydroxystilbene) has been shown to inhibit protein degradation and attenuate atrophy of skeletal muscle fibers in several *in vitro* studies [14–17]. A relatively high dose (400 mg/kg/day) of resveratrol, *in vivo* has been shown to attenuate slow muscle fiber atrophy following hindlimb suspension [18] in rodents. In agreement with these data, we have found that a low dose (12.5 mg/kg/day) of resveratrol [19] had a trend (p=0.06) to blunt fast muscle losses during hindlimb suspension induced muscle wasting. However, we do not know if resveratrol has the potential to improve recovery of muscle after wasting conditions in aging. As muscle regeneration and repair is critically dependent upon adequate proliferation and differentiation of muscle stem cells (satellite cells), it is important to know if resveratrol treatment would improve satellite cell function or prevent nuclear loss (e.g., apoptosis) *in vivo*. This seems plausible because resveratrol has been shown to improve non-cancer cell viability, reduce apoptosis [20,21] and promote stem cell proliferation and cell repair [22,23], but this is cell type and dose dependent. Nevertheless, the function of resveratrol on skeletal muscle stem cell function during muscle repair is less clear. Sirtuin 1, a presumed target of resveratrol has been reported to increase proliferation [24] of skeletal muscle stem cells/myoblasts in culture, whereas resveratrol treatment has been found to increase differentiation of myoblasts rather than proliferation *in vitro* [23]. As resveratrol may function differently on skeletal muscle satellite cells *in vivo* in aging vs. these *in vitro* studies, we tested the hypothesis that resveratrol supplementation would alter satellite cell proliferation and reduce pro-apoptotic signaling to improve muscle regeneration in muscles of aged rats following muscle disuse. This line of inquiry is important because muscles from aged animals recover very little after disuse (26) and this is confirmed in the current study. The findings of our study suggest that resveratrol may modestly improve the apoptotic environment of muscles in aged rodents during reloading following disuse, but it has a limited ability to alter satellite cell proliferation as a mechanism for recovering muscle mass that was lost during the period of disuse.

## Methods

### Animals

Thirty-six male Fisher 344 × Brown Norway rats were obtained from the National Institute on Aging (NIA) colony that is housed at Harlan (Indianapolis, IN). All of these animals were 32 months of age. The animals were housed at 20°C in barrier-controlled conditions under a 12:12 hour light-dark cycle. Proper animal care standards were followed by adhering to the recommendations for the care of laboratory animals as advocated by the American Association for Accreditation of Laboratory Animal Care and by following the policies and procedures detailed in the Guide for the Care and Use of Laboratory Animals as published by the U.S. Department of Health and Human Services and proclaimed in the Animal Welfare Act (PL89-544, PL91-979, and PL94-279). All experimental procedures carried approval by the Institutional Animal Care and Use Committee from the West Virginia University.

### Hindlimb suspension

The animals were randomly assigned to ambulatory cage control (n=12), hindlimb suspended (n=12), or reloaded after suspension (recovery; n=12) groups. The hindlimb suspension lasted fourteen days as previously described [25]. Briefly, orthopedic tape was applied along the proximal one-third of the animals’ tail and then placed through a wire harness attached to a swivel. This was fixed to the top of a specially designed hindlimb suspension cage which provided the rats with 360° of movement around the cage. The forelimbs maintained contact with a grid pattern floor, allowing the animals to move, groom themselves, and obtain food and water freely. The exposed tip of the tail was closely monitored to ensure that it remained pink, indicating that hindlimb suspension did not interfere with blood flow to the tail. The suspension height was monitored and adjusted to prevent contact between the hindlimbs and any supportive surface of the cage. Care was taken so that the suspension angle of the torso of the animals to the cage floor did not exceed 30°. All animals were weighed prior to and following the suspension period to determine total body mass changes. Furthermore, the recovery animals were released from suspension after the fourteen day protocol and provided an additional fourteen days of normal ambulation, at which point they were weighed and sacrificed. 

### Nutritional treatment

Beginning one week prior to suspension, the rats received 1ml of 0.1% carboxymethylcellulose dissolved in distilled water (vehicle treated), and the remaining eighteen rats received 1ml of trans-resveratrol suspended in 0.1% carboxymethylcellulose dissolved in distilled water (resveratrol treated), administered via oral gavage daily throughout the study. Previous studies in our lab [19,26] using resveratrol (12.5 mg/kg/day in the diet, or 50 mg/kg/day by oral gavage) were too low to effectively preserve muscle mass. Therefore, in this study resveratrol was administered at a dose of 125 mg/kg/day in this study in order to increase the likelihood of detecting the effects of this compound on muscle wasting and regeneration. This provided a bolus increase in resveratrol every 24 hours. To ensure resveratrol was also available systemically throughout the day, the resveratrol treated animals were given food pellets that contained 0.05% resveratrol. Vehicle treated animals had an identical diet to the resveratrol treated animals, but their chow did not contain resveratrol. This provided a mechanism to provide a more consistently high level of resveratrol throughout the day. Food and water was provided *ad libitum.*

### Bromodeoxyuridine administration

Immediately following suspension, each of the recovery animals received a 200mg bromodeoxyuridine (BrdU; 5-bromo-2’-deoxyuridine) pellet (21-day release; Innovative Research; Sarasota, FL) in order to analyze myonuclear (satellite cell) proliferation, as BrdU is a thymidine analog which is incorporated into nuclei during DNA synthesis. Upon release from suspension, a small incision was made in the upper back of anesthetized animals whereupon the BrdU pellet was inserted subcutaneously. The incision was closed with a wound clip (MikRon Autoclip 9 mm; Becton Dickinson; Sparks, MD) and the animals were allowed to recover from anesthesia before being returned to their cage. 

### Research design

Muscle data were obtained from both the resveratrol and vehicle-treated animals after hindlimb suspension, or recovery. In addition, two groups of ambulatory non-suspended cage control animals were used. Six cage control animals were examined 21 days after the initiation of the study. These animals acted as controls for the hindlimb suspension groups. A second group of six cage control animals were examined 35 days after the initiation of the study and were used as controls for the recovery groups. Among the suspended animals, six per diet group (i.e., vehicle or resveratrol) were sacrificed 21 days after the initiation of the study (hindlimb suspension) and another six animals per diet group were sacrificed 35 days after the initiation of the study (recovery). The six hindlimb suspension cage control and six recovery cage control animals were also sacrificed at these respective time points.

### Tissue collection

At the end of the 21 and 35 day experimental periods for the hindlimb suspension and recovery groups respectively, the animals were sacrificed while under deep anesthesia (5% Isoflurane/95% Oxygen). The soleus, gastrocnemius, and plantaris muscles were excised from both hind limbs, blotted to remove excess fluid, and individually weighed. The weight of each muscle was normalized to the animals’ body weight and subsequently compared to determine if resveratrol preserved muscle mass. A block obtained from the mid-belly of the muscle was embedded in optimal cutting temperature (OCT) compound, flash frozen in liquid nitrogen cooled isopentane, and stored at -80°C until later analyses. Blood samples were obtained from the left ventricle of each animal via a stainless steel needle (19G1½; Becton Dickinson; Rutherford, NJ). The concentration of resveratrol was determined in the serum samples by high-performance liquid chromatography against known standards (Protea Biosciences, Inc.; Morgantown, WV). Following blood collection, the animals were immediately euthanized by removing the heart with surgical scissors. 

### Resveratrol and metabolites of resveratrol in plasma

Plasma from resveratrol and control animals was analyzed for resveratrol and its metabolites: Trans-Resveratroi-3-0-Sulfate, Trans-Resveratroi-3-0-Giucuronide, and Trans-Resveratroi-4-0-Giucuronide. Plasma was analyzed by Protea Biosciences (Morgantown, WV) using high performance liquid chromatography (HPLC). Quantification of the data was made against standard curves (generated in duplicate) for each metabolite.

### Sirt1 activity

Sirt1 activity was measured in total plantaris muscle homogenates using a fluorescent deacetylase substrate using a commercially available kit (BML-AK555; Enzo Life Sciences Inc.; Farmingdale, NY). Briefly, muscles were homogenized in ice-cold distilled water and the protein concentrations were determined using a DC protein concentration assay (BioRad). The tissue samples were diluted to 2.5 μg/μL using the reagents supplied in the kit. The fluorescent substrate, in conjunction with 100 μM of the co-substrate NAD^+^, was then incubated with 15 μL of each sample for 45 minutes at room temperature (RT) in a ½ volume 96-well white microplate. Once complete, 2mM nicotinamide (a Sirt1 inhibitor) and the provided fluorescent developer were added to each well to halt the reaction and produce a fluorophore which is linearly related to Sirt1 activity. The intensity of the fluorescent signal was detected with an excitation wavelength of 360 nm and an emission wavelength of 460 nm. Data are presented as fluorescent units normalized to the respective milligrams of protein used in each homogenate.

### Ex vivo muscle physiological analyses

Isometric muscle contractile properties were examined in the plantaris muscles from the left leg of all experimental animals. The muscles were placed in an oxygenated Ringer’s solution (137 mM NaCl, 4.7 mM KCl, 3.4mM CaCl_2_, 1.2mM MgSO_4_, 1 NaH_2_PO_4_, and 112 D-glucose), which was maintained at 20°C. The Ringer’s solution was aerated with 95% O_2_ and 5% CO (pH 7.4). The distal end of the muscle was attached to a stationary plexiglass plate, and the proximal end fixed to the lever arm of a 300C dynamometer (Aurora Scientific, Aurora Ontario, Canada). The muscles were stimulated by passing a constant current through platinum plates positioned on each side of the muscle. *Ex vivo* isometric twitch and tetanic contractions were obtained using a Constant Current/Constant Voltage Stimulator (Aurora Scientific) that provided DC-square wave signals at a stimulation current of 12 Volts, with a 200 µs pulse width. Muscles were adjusted to the optimal muscle length (L_o_) by a micromanipulator that controls the base position of the electrode clamp. L_o_ was established as the muscle length that produced maximal isometric twitch tension and was periodically checked by the same procedure throughout each experiment to maintain this length. Force-frequency isometric force records were obtained by stimulating the muscle at 10, 20, 40, 50, 75 and 100 Hz, with 3 minutes of rest between each contraction. Physiological contractile measures included peak isometric twitch force (PT), time to peak twitch contraction tension (CT), and the ½ relaxation time of twitch contraction (½ RT), as previously described [27]. Following isometric contractions, the muscles remained in the oxygenated Ringer’s solution for 5 minutes prior to the repeated stimulation fatigue protocol. Muscle fatigue was assessed by stimulating the muscle at 40Hz for 3 minutes utilizing a duty cycle of 330 ms of stimulation followed by 660 ms of rest [28]. The fatigue index was calculated as the force from the last contraction expressed as a percentage of the force obtained on the first contraction. The contractile and fatigue measurements were analyzed off line using commercial software (DMI, Aurora Scientific).

### Fiber characteristics

Muscle fiber type and cross-sectional area (CSA) analyses were performed in the plantaris muscles. Frozen tissue cross sections measuring 10-μm thick were cut from the mid-belly of the muscles, mounted on charged microscope slides (Fisher Scientific; Pittsburgh, PA), and stored at -80°C. For analysis, the slides were air-dried 30 minutes, washed in PBS, and incubated for 30 minutes at RT with 4% bovine serum albumin in PBS. The tissue sections were incubated overnight at 4°C with antibodies directed against laminin (MAB1914; Millipore; Temecula, CA) to visualize the basal lamina of each muscle fiber, as well as one of the following individual myosin heavy chain (MyHC) antibodies: BA-D5 (for MyHC I; Developmental Studies Hybridoma Bank; Iowa City, IA), SC-71 (for MyHC IIA; DSHB), and BF-F3 (for MyHC IIB; DSHB) in order to determine both the whole muscle composition and mean cross-sectional area (CSA) for each respective fiber type. The next day, the slides were washed in PBS and incubated 1 hour at 37°C with secondary antibodies of donkey anti-rat rhodamine conjugate (712-025-150; Jackson ImmunoResearch Laboratories; West Grove, PA) to visualize the basal lamina, and Alexa Fluor 488 (A10680; Invitrogen; Eugene, OR) to visualize the MyHC expression in the muscle fibers. The sections were mounted with 4', 6-diamidino-2-phenylindole (DAPI) (Vectashield Mounting Medium; Vector Laboratories; Burlingame, CA) to visualize the nuclei. Four images from non-overlapping regions of each tissue cross-section stained for individual MyHC fibers were used to determine both fiber type proportions and respective CSA’s. All images were taken with a Zeiss LSM 510 Meta confocal microscope (Carl Zeiss Microimaging Inc.; Thornwood, NY) at a magnification of 20X. Mean fiber CSA of respective fiber types was determined by planimetry and calculated by the ImageJ software (NIH; Frederick, MD). Fiber types are expressed as a proportion of MyHC-positive fibers to total fibers for each individual MyHC examined. The percentage of type IIX MyHC fibers was calculated from fibers that were negative to type I, IIA, and IIB MyHC antibodies. 

### Western blotting

Western blots were used to determine the relative amount of proteins in apoptotic signaling pathways following hindlimb suspension and recovery. Approximately seventy-five micrograms of muscle was homogenized in ice-cold modified RIPA buffer (50mM Tris, 1% NP-40, 150mM NaCl, 1mM EDTA, 0.1% SDS, 0.5% Na-deoxycholate; pH: 7.4) containing protease and phosphatase inhibitors. The muscle homogenates were centrifuged at 1,000*g* for 5 minutes at 4°C, and the resulting supernatant was collected. The protein content of the samples was measured using the DC protein assay kit (BioRad; Hercules, CA). Thirty-five micrograms of protein was loaded into each well of a 4-12% gradient polyacrylamide gel (Invitrogen; Carlsbad, CA) and separated by routine sodium dodecyl sulfate-polyacrylamide gel electrophoresis (SDS-PAGE) for 2 hours at 135V. The proteins were then transferred to a nitrocellulose membrane for 2 hours at 35V. Non-specific protein binding was blocked by incubating the membranes in 5% nonfat milk in Tris-buffered saline containing 0.05% Tween 20 (TBST) at RT for 1 hour. Membranes were incubated overnight at 4°C on a rocking table with primary antibodies (1:1,000) directed against Bcl-xL (#2762; Cell Signaling Technology; Boston, MA), Bax (#2772; Cell Signaling) Bcl-2 (#2876; Cell Signaling), cleaved caspase 3, (#9664; Cell Signaling), cleaved caspase 9 (#9507; Cell Signaling), AMPK (#5832; Cell Signaling), pAMPK (#2535; Cell Signaling), Sirt1 (#9475; Cell Signaling) and PGC1(#sc-13067; Santa Cruz Biotech; Dallas, TX). Furthermore, a primary antibody (1:2,000) was directed against GAPDH (ab8245; Abcam; Cambridge, MA) for use as a loading control. The following day, the membranes were washed in TBST and incubated in appropriate dilutions of secondary antibodies (diluted in 5% non-fat milk) conjugated to horseradish peroxidase for one hour at RT on a rocking table. The signals were developed using a chemiluminescent substrate (Lumigen; Southfield, MI) and visualized by exposing the membranes to x-ray film (BioMax MS-1; Eastman Kodak, Rochester, NY). Digital records of the bands were captured using a Kodak 290 camera and quantified using one-dimensional analysis software (Eastman Kodak) as optical density × band area, expressed in arbitrary units relative to appropriate loading controls. 

### Identification of apoptotic nuclei

Frozen tissue 10-μm thick cross sections were obtained from the plantaris muscles and mounted on charged microscope slides (Fisher Scientific; Pittsburgh, PA). Fluorescent labeling of terminal dUTP nick-end labeling (TUNEL) with lamina allowed the detection of apoptotic nuclei in the muscle sections. This was accomplished using a slight modification to the method previously reported for our lab [29]. Briefly, the tissue sections were air dried, fixed with 4% paraformaldehyde, and permeabilized with 0.2% Triton X-100 in PBS for 30 minutes each at room temperature. The tissues were incubated overnight at 4°C in a rat anti-lamina monoclonal antibody (Millipore). Sections were incubated the following day with donkey anti-rat rhodamine conjugated secondary antibody (Jackson ImmunoResearch), along with the TUNEL reaction mixture (11-684-795-910; Roche Diagnostics; Indianapolis, IN) in a humidified chamber at 37°C for 1 hour in the dark. The exclusion of the TdT enzyme in the TUNEL reaction mixture on one of the tissue sections on each slide was included as a negative control. The sections were mounted with DAPI (Vectashield) in order to visualize nuclei and viewed under a Zeiss LSM 510 Meta confocal microscope (Carl Zeiss Microimaging Inc.; Thornwood, NY). The number of TUNEL and DAPI-positive nuclei that were immediately adjacent to, or beneath the basal lamina were counted. The data are expressed as an apoptotic index, calculated as the percentage of TUNEL-positive nuclei out of the total myonuclei (i.e., DAPI-positive nuclei) pool. The apoptotic index was determined from four non-overlapping regions of each tissue cross section visualized with a 20X objective. 

### BrdU detection

Frozen tissue cross sections measuring 10-μm thick from the plantaris muscle were mounted on charged microscope slides (Fisher Scientific; Pittsburgh, PA). Briefly, tissue sections were air dried, washed in PBS, fixed in methanol: acetone (1:1) at -20°C for 5 minutes, and permeabilized with 0.4% Triton X-100 in PBS. Sections were then denatured, blocked with serum and incubated overnight in a biotinylated mouse anti-BrdU primary antibody (HCS30, EMD Biosciences, Inc., La Jolla, CA). On the following day, the sections were incubated with a secondary antibody of Fluorescein Avidin DCS (A-2011; Vector Laboratories, Inc.; Burlingame, CA) and subsequently blocked in normal goat serum. They were then incubated overnight in a rat anti-lamina monoclonal (Millipore) primary antibody. The tissue sections were incubated the following day with a donkey anti-rat rhodamine conjugated (Jackson ImmunoResearch) secondary antibody in a humidified chamber at 37°C for 1 hour in the dark. Finally, the sections were mounted with 4',6-diamidino-2-phenylindole (DAPI)-containing mounting medium (Vectashield) in order to visualize nuclei. Immunofluorescence was visualized with a Zeiss LSM 510 Meta confocal microscope (Carl Zeiss). Images were taken from four non-overlapping regions of each tissue section with a 20X objective. 

### Statistical analysis

All results are reported as means ± SD. Differences in means between groups were determined by multiple analysis of variance (MANOVA) Hotelling's T-Square test. Bonferroni post hoc analyses were subsequently performed between significant means. A *P*-value that was <0.05 was considered significant.

## Results

### Body weight

**Figure 1:**
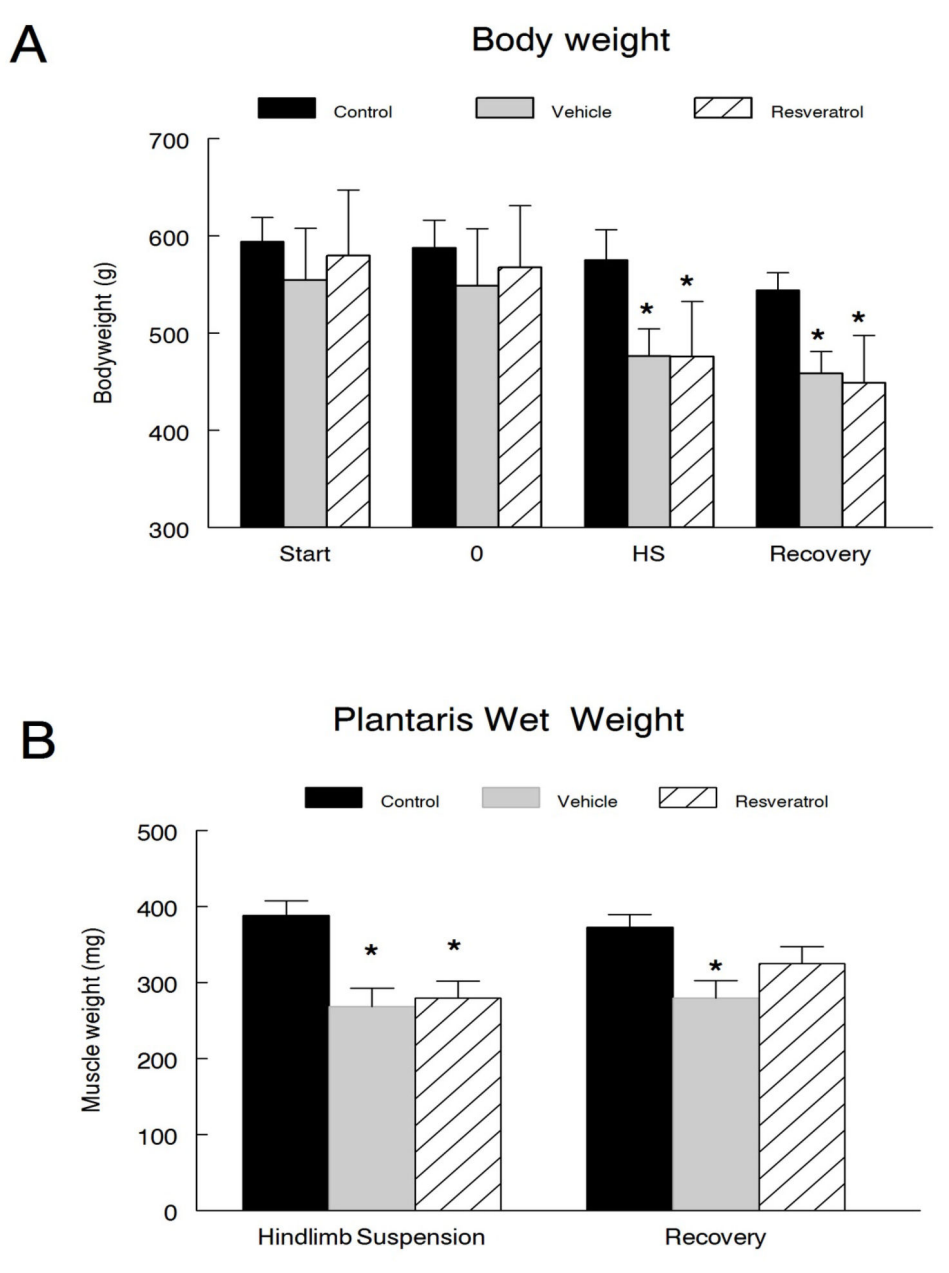
Body and muscle wet weights. *A*) Bodyweight measurements of all animals were taken immediately prior to the start of the one-week pretreatment period (Start), prior to suspension (0), following hindlimb suspension (HS), and again following the reloading period recovery in cage control (n=12), vehicle-treated (n=12) or resveratrol-treated (n=12) animals. *B*. Plantaris muscle wet weights were obtained following HS or R. * *P*<0.05 *vs*. cage control.

We measured the bodyweight of all animals at the beginning of the one-week pretreatment period (Start), immediately prior to suspension (Day 0), following hindlimb suspension, and following the recovery period. There were no differences in bodyweight between any of the groups at the beginning of the experimental protocol. Furthermore, no significant changes in bodyweight were observed in the cage control animals at any of the time points. A significant loss of bodyweight occurred in all of the suspended animals as compared to the first day (Start) of the experiment, with decreases of 24.6% and 18.0% observed in the vehicle and resveratrol treated groups, respectively. These significant losses continued into the recovery period as well, with vehicle and resveratrol treated animals losing an additional 3.7% and 5.6% of body mass, respectively, between the end of hindlimb suspension and the end of the recovery period. However, resveratrol was unable to attenuate any of these bodyweight reductions, as there were no significant differences observed between the vehicle and resveratrol treated animals at any of the individual time points of the study (Figure **1A**).

### Muscle wet weight

Upon sacrifice, the plantaris muscle wet weights were examined to assess changes in muscle mass following the suspension and recovery periods. As expected, hindlimb suspension induced significant decreases (*P*<0.001) in absolute muscle mass in both the vehicle and resveratrol treated groups as compared to the hindlimb suspension control animals (Figure **1B**). However, there were no differences between the vehicle and the resveratrol treated animals. Following the recovery period, the wet weights from both the vehicle treated animals was still suppressed but the muscle weight of the resveratrol treated animals fully recovered to that of the control animals’ muscle mass. 

### Muscle contractile characteristics

**Figure 2:**
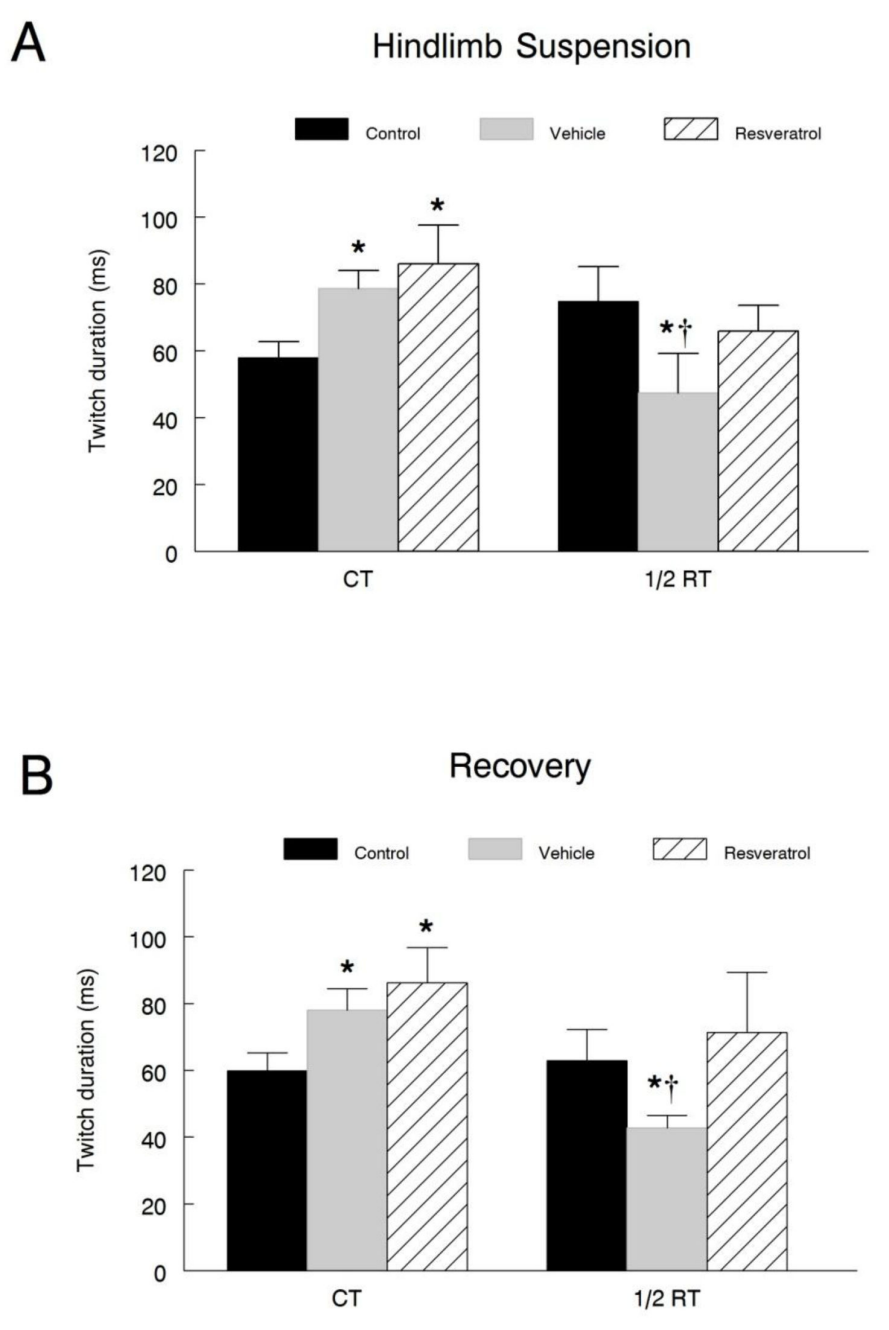
Plantaris muscle twitch characteristics. Muscle twitch contractile properties including CT and ½ RT were analyzed in the plantaris muscles of both the hindlimb suspension (**A**) and recovery (**B**) groups. Each data point represents the average of three measures. * *P*<0.05 *vs*. cage control; † *P*<0.05 vehicle *vs*. resveratrol.

Plantaris muscles were attached to a force transducer in oxygenated Ringers solution, to determine various muscle contractile properties following hindlimb suspension and recovery. Following hindlimb suspension, there was a significant increase (*P*<0.001) in CT during a twitch contraction in both the vehicle (78.6 ± 5.3 ms) and resveratrol (86.2 ± 10.6 ms) treated groups as compared to the cage controls (38.7 ± 6.8 ms). However, resveratrol was unable to attenuate this increase (Figure **2A**). In the recovery group, the CT of both the vehicle (78.0 ± 6.4 ms) and resveratrol (86.2 ± 10.6 ms) animals was significantly greater than that of the recovery cage control (62.0 ± 8.0 ms) animals (Figure **2B**). There were no differences observed when comparing the CT of the vehicle and resveratrol treated animals. 

Next, we examined the ½ RT during a twitch contraction. In the hindlimb suspension group, the ½ RT was significantly shorter in the vehicle treated (47.4 ± 11.8 ms) animals compared to either the resveratrol (65.8 ± 7.8 ms) or cage control (69.8 ± 9.6 ms) groups (Figure **2A**). Moreover, there were no differences between the resveratrol and cage control groups at this time point. This same trend also continued into the recovery period (Figure **2B**). While there were no differences in the ½ RT between the resveratrol (71.3 ± 18.1 ms) and cage control (61.2 ± 13.0 ms) groups, the ½ RT of the vehicle treated (42.7 ± 3.8 ms) group was significantly shorter than both of these two groups. 

**Figure 3:**
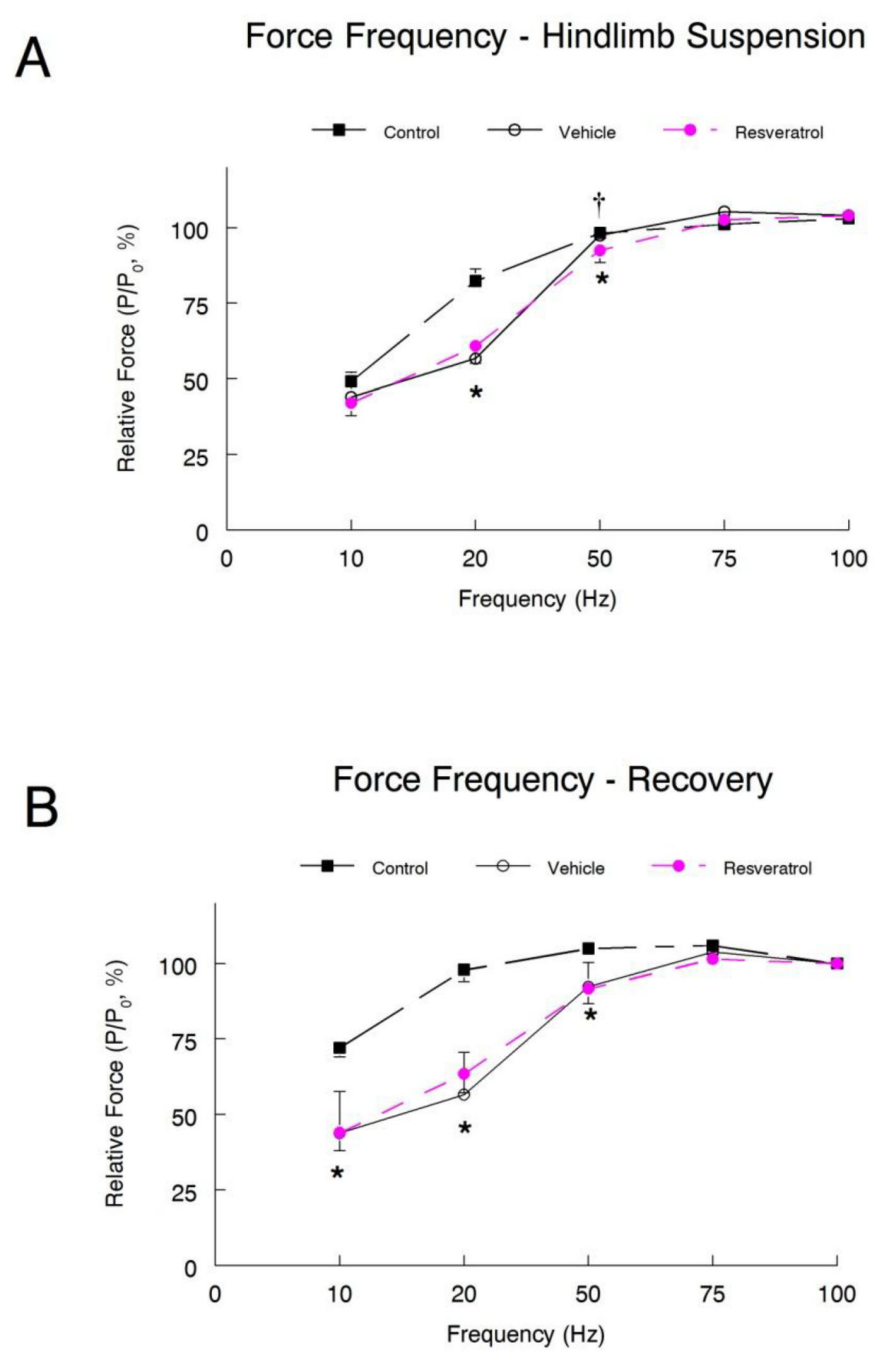
Force-frequency relationship. A force-frequency curve was plotted for both the hindlimb suspension (**A**) and recovery (**B**) groups. Each force-frequency measurement was made three times with a minimum of 5 minutes rest between each contraction, and the average of the three trials was recorded for each animal. * *P*<0.05 vehicle and resveratrol *vs*. cage control; † *P*<0.05 vehicle *vs*. resveratrol.

Lastly, we plotted the force-frequency curve for the hindlimb suspension and recovery groups. In the hindlimb suspension animals, a frequency of 20 Hz produced a significant rightward shift in both the vehicle and resveratrol treated groups (Figure **3A**). At 50 Hz, there remained a significant rightward shift in the resveratrol animals curve compared to both the cage control and vehicle treated animals. In contrast, there were no differences observed between the vehicle treated and cage control groups. At 75 Hz, both the vehicle and resveratrol treated groups reached their peak relative forces. 

In the recovery animals, the force frequency curves from both the vehicle and resveratrol treated groups were significantly shifted to the right of the cage control curve at 10, 20, and 50 Hz (Figure **3B**). However, the force-frequency curves of the vehicle and the resveratrol groups did not differ. The force record at 75 Hz in the resveratrol treated animals had a significant rightward shift as compared to the cage controls. 

### Muscle fatigue characteristics

**Figure 4:**
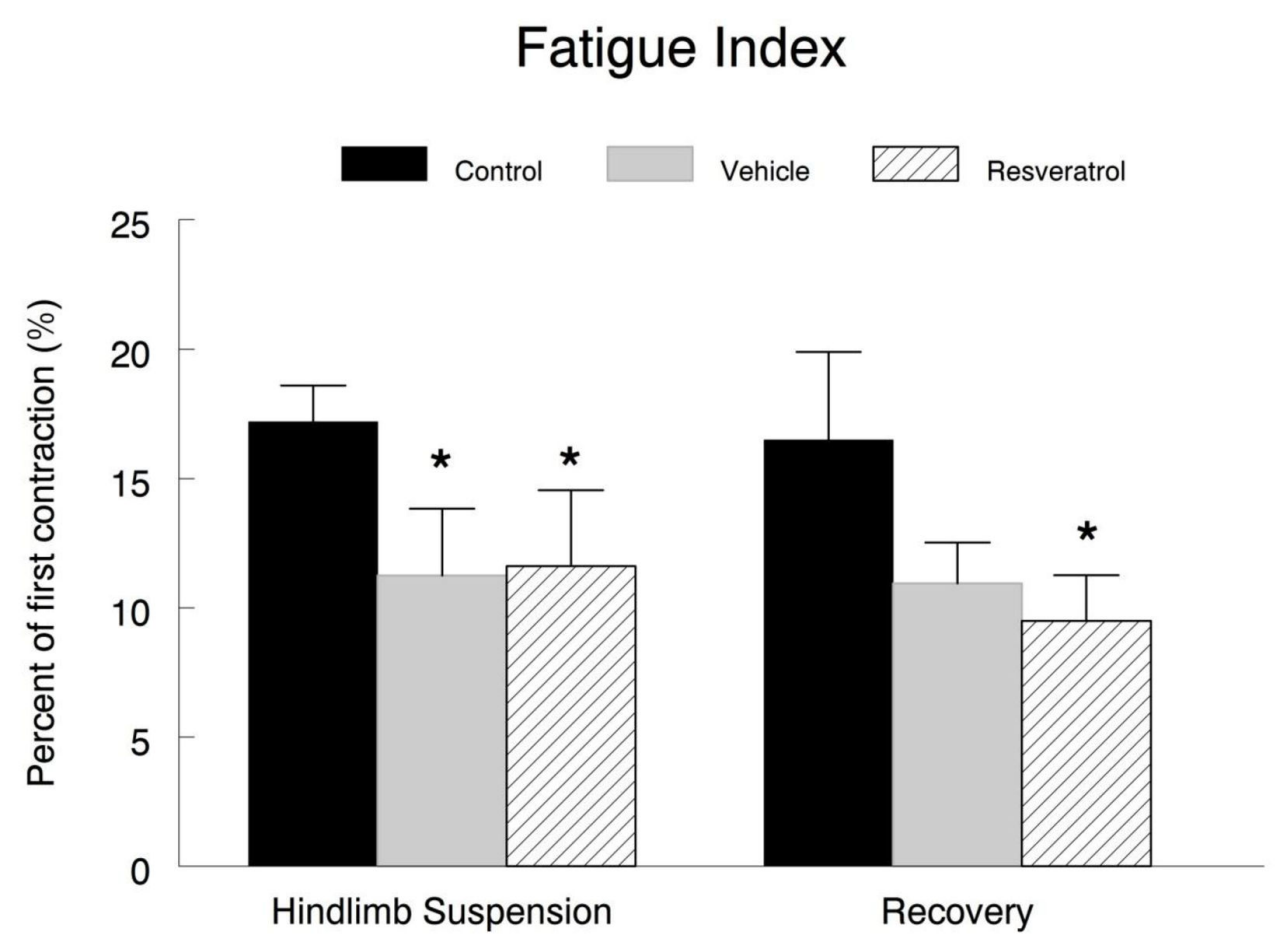
Fatigue index. Plantaris muscle fatigability was expressed as a fatigue index, and calculated as the percent of the initial force ( average of the first three contractions, divided by the force at the end of the fatigue protocol (an average of the force generated in the final three contractions) in a series of 128 consecutive contractions. * *P*<0.05 *vs*. cage control; † *P*<0.05 vehicle *vs*. resveratrol.

The fatigue index was examined in order to determine if resveratrol had improved the muscles fatigue tolerance during a series of contractions. As expected, hindlimb suspension elicited a significant decrease in the muscles’ resistance to fatigue. Both the vehicle (11.3 ± 2.6%) and the resveratrol (11.6 ± 2.9%) treated animals had significantly reduced fatigue indexes as compared to the cage control (16.6 ± 1.8%) group (Figure **4**). However, there were no differences observed between the resveratrol and the vehicle treated animals. In the recovery animals, the vehicle treated animals had a similar fatigue index (10.9 ± 1.6%) as the resveratrol (9.5 ± 1.8%) fed group; however the fatigue index of the resveratrol group was significantly lower than the cage control (14.8 ± 4.8%) group after reloading (Figure **4**). 

### Sirt1 activity

Plasma resveratrol and it’s metabolites were determined by HPLC analysis. The plasma level of resveratrol, in resveratrol-treated animals averaged 400.3 ± 69.9 ng/ml at the point of sacrifice. There were no differences between the animals in the hindlimb suspension or control groups. Metabolites of resveratrol were similarly very high in the resveratrol treated animals (Table S1). While there were no traces of resveratrol in the plasma of any of the vehicle treated animals, there was a detectible about of several resveratrol metabolites in the plasma of vehicle treated animals (Table S1) suggesting that perhaps the control diet contained small but detectable levels of resveratrol which had been metabolized by the vehicle-treated animals. 

**Figure 5:**
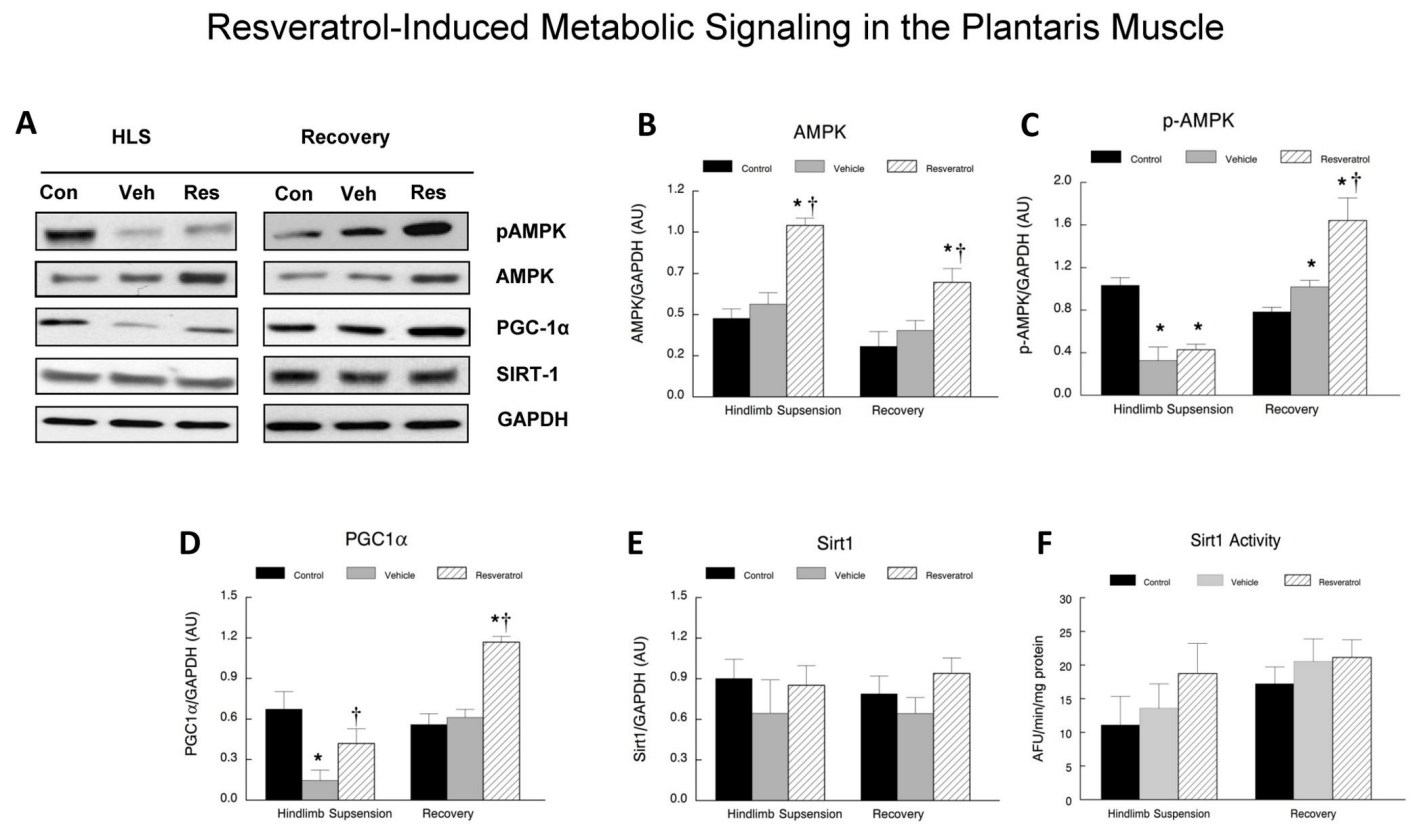
Resveratrol-induced metabolic signaling in the plantaris muscle. **A**. Western blots were conducted for plantaris muscles after hindlimb suspension or following recovery from hindlimb suspension. The protein content was measured in total plantaris muscle homogenates via immunoblotting. Total muscle protein lysates were separated on a 4-12% gradient polyacrylamide gel by routine SDS-PAGE. The proteins were electroblotted to nitrocellulose membranes and the signals were developed by chemiluminescence. The proteins included phosphorylated (activated) AMPK, total AMPK, PGC1α, and Sirt1. GAPDH was used as an internal loading control. Con, control; Veh, vehicle-treated; Res, resveratrol treated. **B**-**E**. The digital images of the western blots were quantified as optical density x band area using ImageJ software and normalized to GAPDH, which was used as the loading control for each lane. The data include: **B**, total AMPK; **C**. phosphorylated AMPK (pAMPK); D. PGC1α; and E. Sirt1. The data are expressed as the protein signal to the GAPDH signal and are reported as mean ± SEM in arbitrary units. A minimum of three western blots were completed for each protein, and the data were averaged for each animal. **F**. Sirt1 enzyme activity was determined fluorometrically in plantaris muscle homogenates. Data are expressed as arbitrary fluorescent units (AFU)/µg protein. * *P*<0.05 *vs*. cage control; †*P*<0.05 vehicle *vs*. resveratrol.

The relative activity of Sirt1 in the plantaris muscles of all of the animals was assessed using a fluorometric assay. In the hindlimb suspension animals, there were no significant differences in Sirt1 activity between any of the groups, although there was a trend towards higher levels in the resveratrol treated animals (Figure **5**). This trend continued with the recovery groups as well, although the differences between the groups were less than it was in the hindlimb suspension animals. This was due to a slight elevation in Sirt1 activity in the muscles of both the recovery control and the vehicle treated animals as compared to their respective hindlimb suspension groups. 

Resveratrol treatment elevated AMPK and pAMPK abundance in the plantaris of resveratrol-fed animals in the recovery group as compared to the vehicle treated or control animals (Figure **5**). Sirt1 protein abundance was not improved by resveratrol treatment (Figure **5**). However, resveratrol, appeared to increase the protein abundance of PGC1α, a downstream target of Sirt1, in the plantaris muscles of animals in the recovery group fed resveratrol, as compared to vehicle treated animals. We did not determine if mitochondrial biogenesis had been altered by resveratrol treatment in the muscles of these animals, however, this seems unlikely since the fatigue characteristics of the muscles was not improved by resveratrol treatment. 

### Myosin heavy chain profile

**Figure 6:**
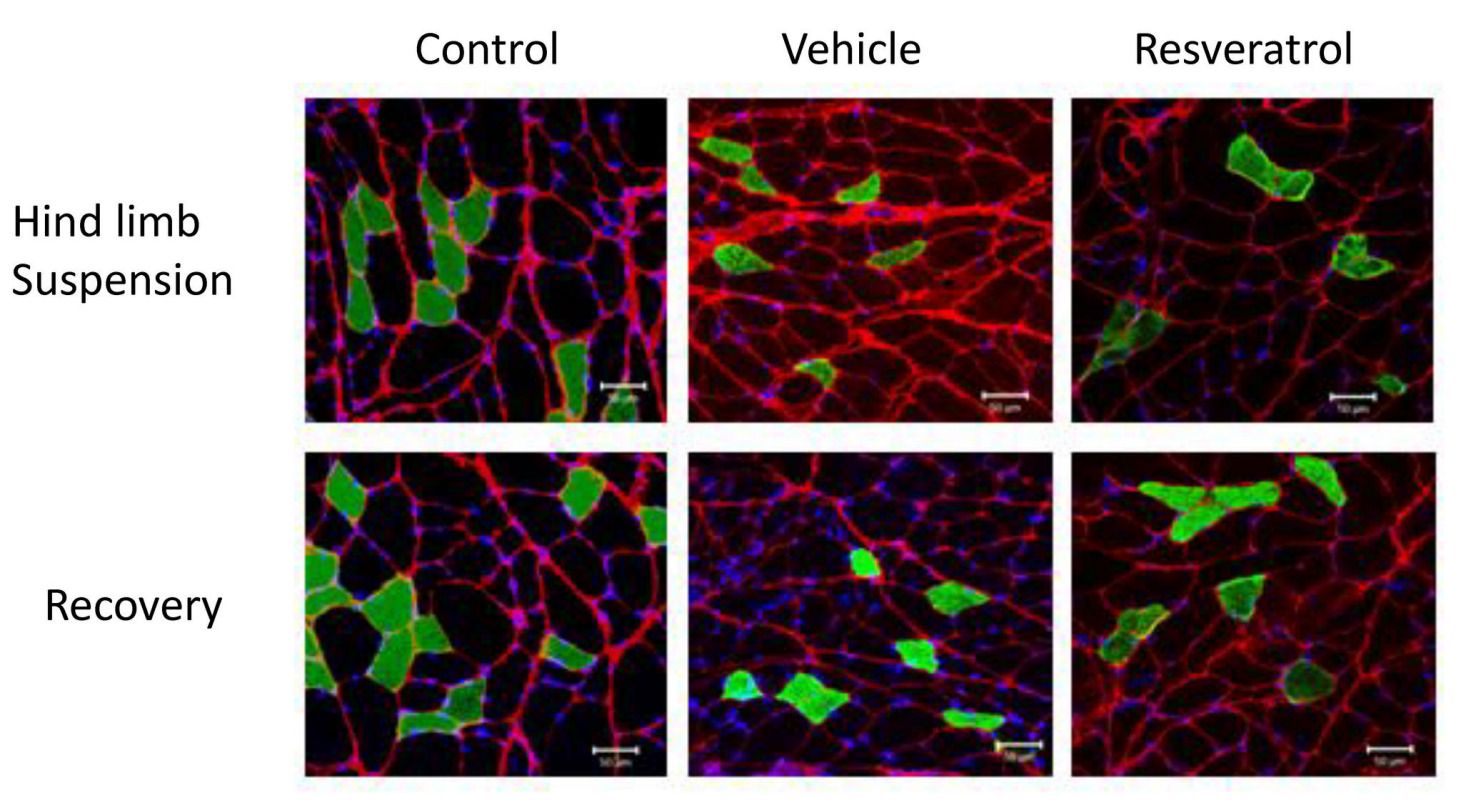
Muscle fiber immunocytochemistry. Frozen tissue cross sections were incubated overnight at 4°C with antibodies directed against the basal lamina (red) and myosin heavy chain I, IIA, or IIB (green). The sections were counterstained with DAPI (blue) to identify the nuclei. Digital images were taken with a confocal microscope. This figure provides a representative example of a section stained for type I myosin heavy chain that was used to measure fiber type composition and CSA analyses for this fiber type in both the hindlimb suspension and recovery groups. Green fibers are positive for type I myosin heavy chains. Scale bars represent 50 µm. * *P*<0.05 *vs*. cage control; † *P*<0.05 vehicle *vs*. resveratrol.

**Figure 7:**
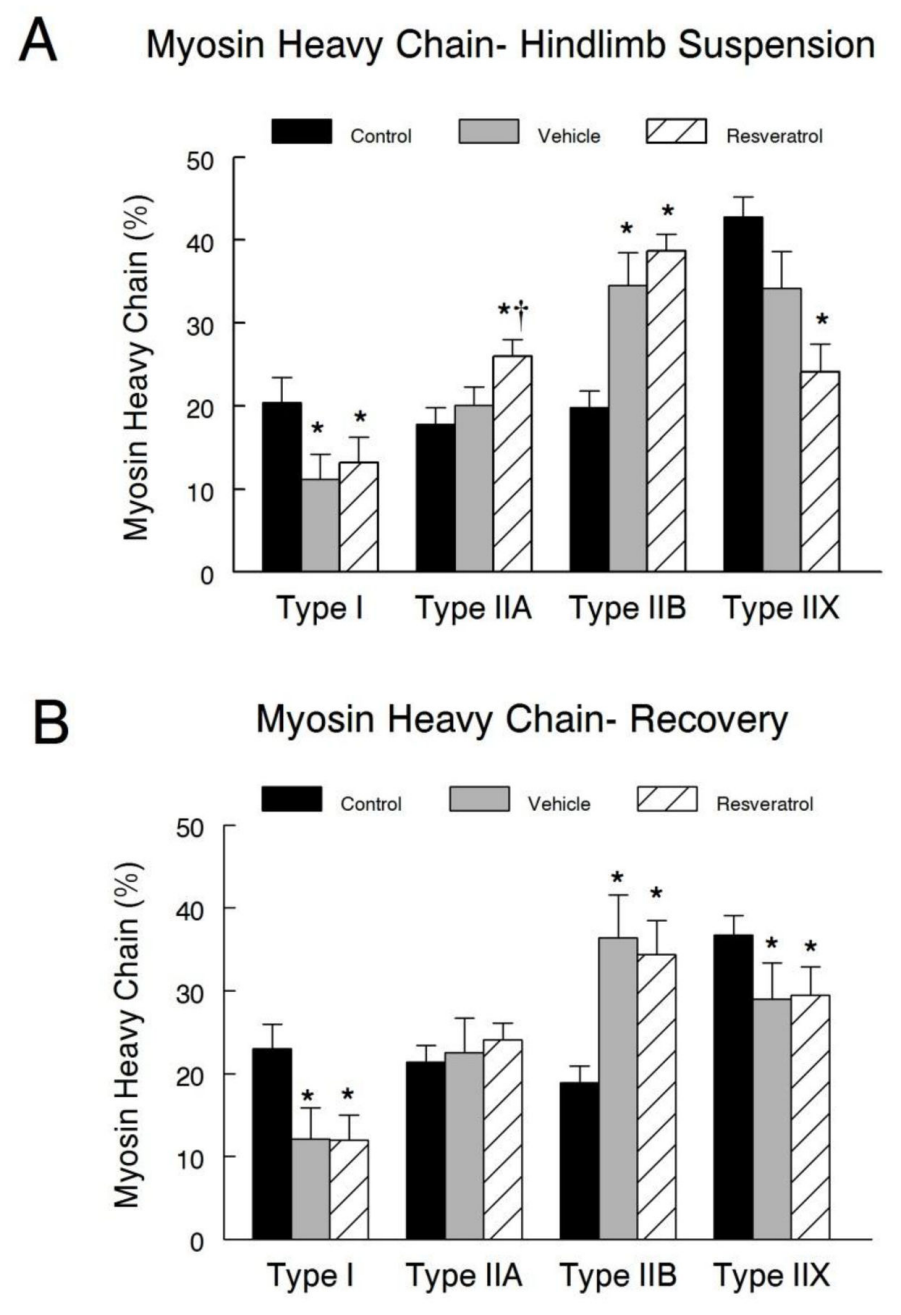
Muscle fiber myosin heavy chain composition and morphology. Immunocytochemical stained plantaris muscle fibers for myosin heavy chains, from control, vehicle-treated and resveratrol-treated rats were analyzed for their myosin heavy chain fiber composition in both the hindlimb suspension (**A**) and recovery (**B**) groups. * *P*<0.05 vs. cage control.

The myosin heavy chain expression was identified in the plantaris muscles fibers by immunocytochemistry (Figure **6**). This provided both an index of fiber type and also fiber size. Typically, hindlimb suspension results in a shift from type I to type II fibers. To determine if resveratrol reduced this fiber type shift following hindlimb suspension or recovery, we examined the myosin heavy chain (MyHC) profile of the plantaris muscles. Following hindlimb suspension, the relative percentage of type I MyHC fibers was significantly reduced in both of the groups subjected to suspension. However, there were no differences between MyHC composition in the vehicle and the resveratrol treated animals, although the mean percentage was slightly higher in the resveratrol group (Figure **7A**). The resveratrol treated animals in the hindlimb suspension group, had a greater percentage of type IIA MyHC fibers, than either the vehicle treated suspended animals, or cage control animals. Suspension significantly increased the percentage of type IIB fibers, although resveratrol had no effect on changes in this fiber type compared to the vehicle treatment. In contrast, the percentage of type IIX MyHC fibers was significantly reduced after suspension in the resveratrol treated animals from that of the control muscles. However, there were no significant differences between the two suspended groups.

The percentage of type I fibers remained significantly less in the animals in the recovery group that had previously been suspended. However, there was an increase in the percentage of type I fibers in the vehicle treated animals to where the resveratrol treated animals no longer had a greater proportion of this fiber type (Figure **7B**). While the percentage of type IIB fibers remained significantly elevated in both the vehicle and the resveratrol treated animals in the recovery group as compared to the control group, there remained no differences between these treatment groups. There was no difference between the percentage of type IIX MyHC fibers in the recovery group of resveratrol and the vehicle treated animals. 

### Fiber size

**Figure 8:**
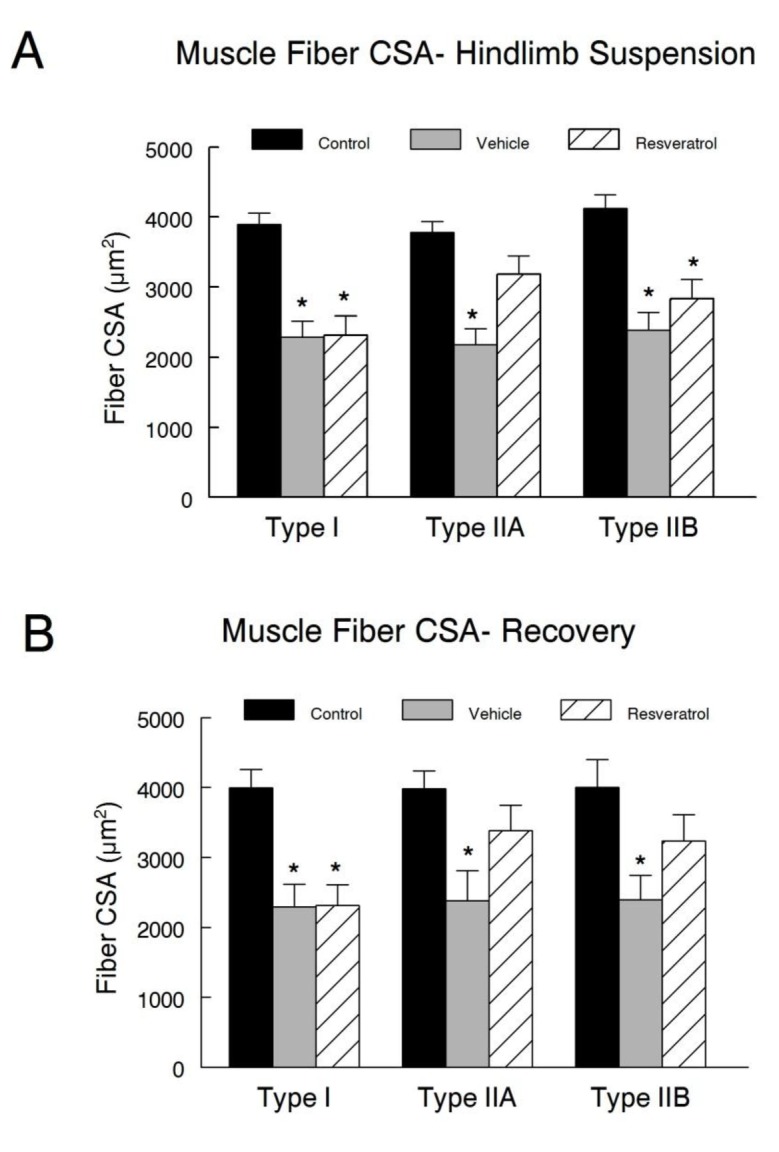
Muscle fiber cross sectional area. Mean fiber cross sectional area (CSA) measurements were obtained from a minimum of 500 fibers in each muscle for plantaris muscle fibers that expressed type I, IIA, or IIB myosin heavy chains in hindlimb suspension (**A**) and recovery (**B**) groups. Four images from non-overlapping regions of each tissue cross-section stained for individual MyHC fibers were used for muscle fiber CSA measures. All images were taken with a Zeiss LSM 510 Meta confocal microscope at a magnification of 20X. Mean fiber CSA of respective fiber types was determined by planimetry. * *P*<0.05 vs. cage control.

Muscle fiber CSA was determined by planimetry in type I, IIA, and IIB MyHC fibers (Figure **6**). As expected, hindlimb suspension induced significant muscle atrophy in all of the fiber types examined. There were no differences in mean type I fiber CSA between the vehicle and resveratrol treated animals after hindlimb suspension, although both hindlimb suspension treatment groups had significantly smaller fibers than the cage control animals. In type IIA fibers, only the vehicle treated animals had myofibers with mean CSA’s significantly smaller than that of the cage control animals (Figure **8A**). The mean fiber CSA from the resveratrol treated animals in the hindlimb suspension group was not different than the cage control animals, indicating that resveratrol partially preserved muscle fiber area during hindlimb suspension in this particular fiber type. Type IIB MyHC fibers CSA was significantly smaller in both the vehicle and the resveratrol treated groups as compared with the fiber CSA from cage control animals. However, no significant differences were noted between type IIB fiber CSA in the vehicle and resveratrol treated groups following hindlimb suspension. 

Animals in the recovery group had a similar fiber type pattern as was found in muscles from the hindlimb suspension animals (Figure **8B**). This suggests that there was no substantial return to control levels in fiber type distribution during the recovery period. Specifically, type I fibers CSA remained significantly smaller in both the vehicle and resveratrol treated animals as compared to the cage controls. Mean type IIA fiber CSA was significantly reduced in the vehicle treated animals as compared to the cage control animals. While the mean CSA of the resveratrol treated animal’s fibers were not statistically different than the vehicle treated animals, the reduction in myofiber size from control levels appeared to be partially attenuated with resveratrol treatment, as these differences did not reach significance. Moreover, although the mean type IIB fiber CSA remained significantly reduced in the vehicle treated animals, the mean CSA of the resveratrol treated animals somewhat recovered and so that fiber CSA was no longer statistically smaller than the control fibers. 

### BrdU incorporation

**Figure 9:**
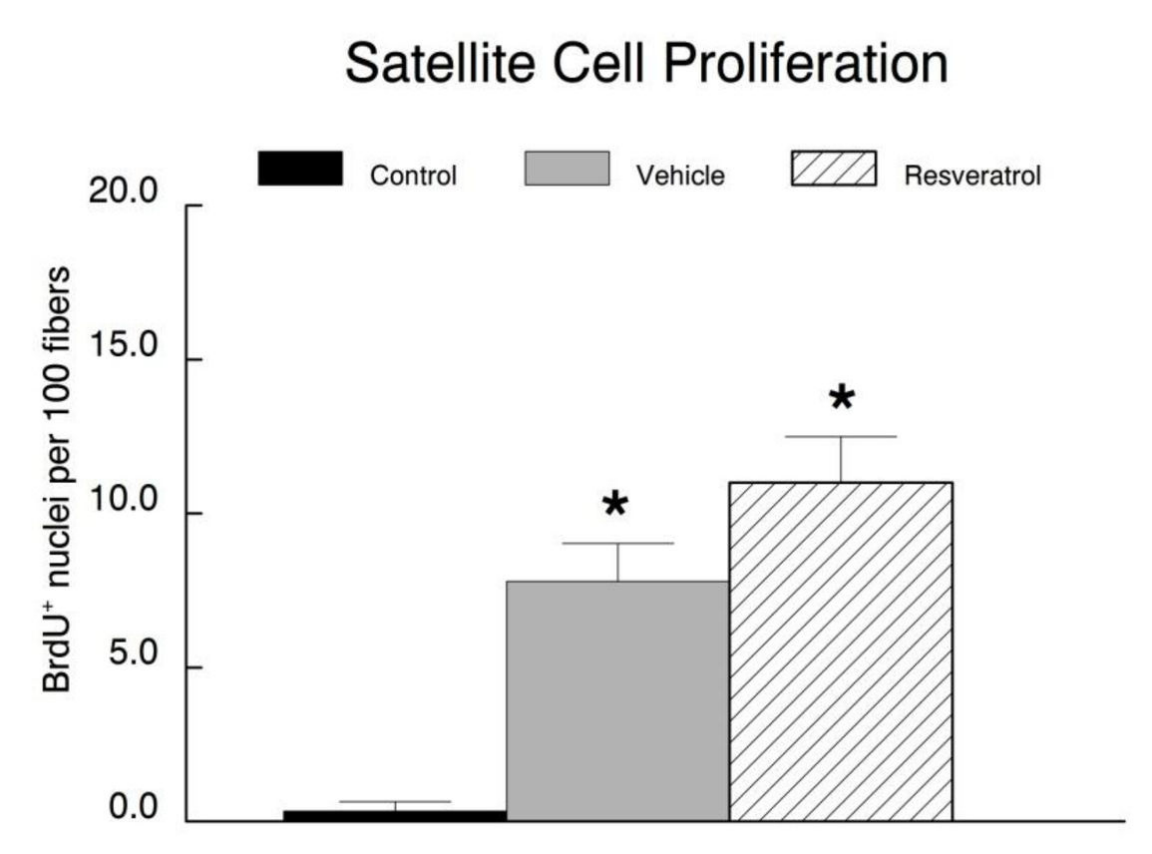
Proliferation of satellite cells. In order to analyze satellite cell proliferation during the reloading period, a time-released BrdU pellet was inserted into the recovery animals at the point that they were released from suspension. BrdU is a thymidine analogue and it was incorporated into muscle nuclei (satellite cells) that divided. A. A representative section from the plantaris of a resveratrol-treated animal that was reloaded for 14 days following 14 days of hindlimb suspension. The tissue was stained with DAPI to identify all of the nuclei, an anti-BrdU antibody to identify nuclei that had proliferated, and an anti-laminin antibody to identify the basal lamina. The overlay shows the three fluorescent images superimposed. The expanded insert shows examples of BrdU positive nuclei that are co-localized to the DAPI stained myonuclei over the basal lamina (white arrows). Other examples of non-specific (green) staining not on nuclei are evident, but they were not included in the quantification of BrdU positive nuclei. B. BrdU incorporation in muscle nuclei (i.e., proliferated satellite cells) is expressed as the number of BrdU-positive nuclei per one hundred myonuclei. * *P*<0.05 *vs*. cage control.

A BrdU pellet was implanted subcutaneously at point that hindlimb suspension was removed, in each of the recovery animals to identify satellite cells that had proliferated during the regenerative period following hindlimb suspension. BrdU-positive nuclei were expressed per 100 myonuclei. BrdU incorporation was very low in muscles of the cage control animals (0.34 ± 0.61). Presumably, there was minimal stimulus to activate any available satellite cells from quiescence under these homeostatic conditions (Figure **9**). It was not surprising that there was a significant increase in BrdU incorporation in both the vehicle (7.8 ± 3.2) and resveratrol (11.0 ± 3.5) treated animals during the recovery period, as satellite cells were activated upon reloading, presumably in an attempt to recover the muscle mass lost during hindlimb suspension. While there was a trend for slightly more BrdU positive nuclei in resveratrol treated muscles, this was not significantly different from the vehicle group. 

### Apoptotic signaling

**Figure 10:**
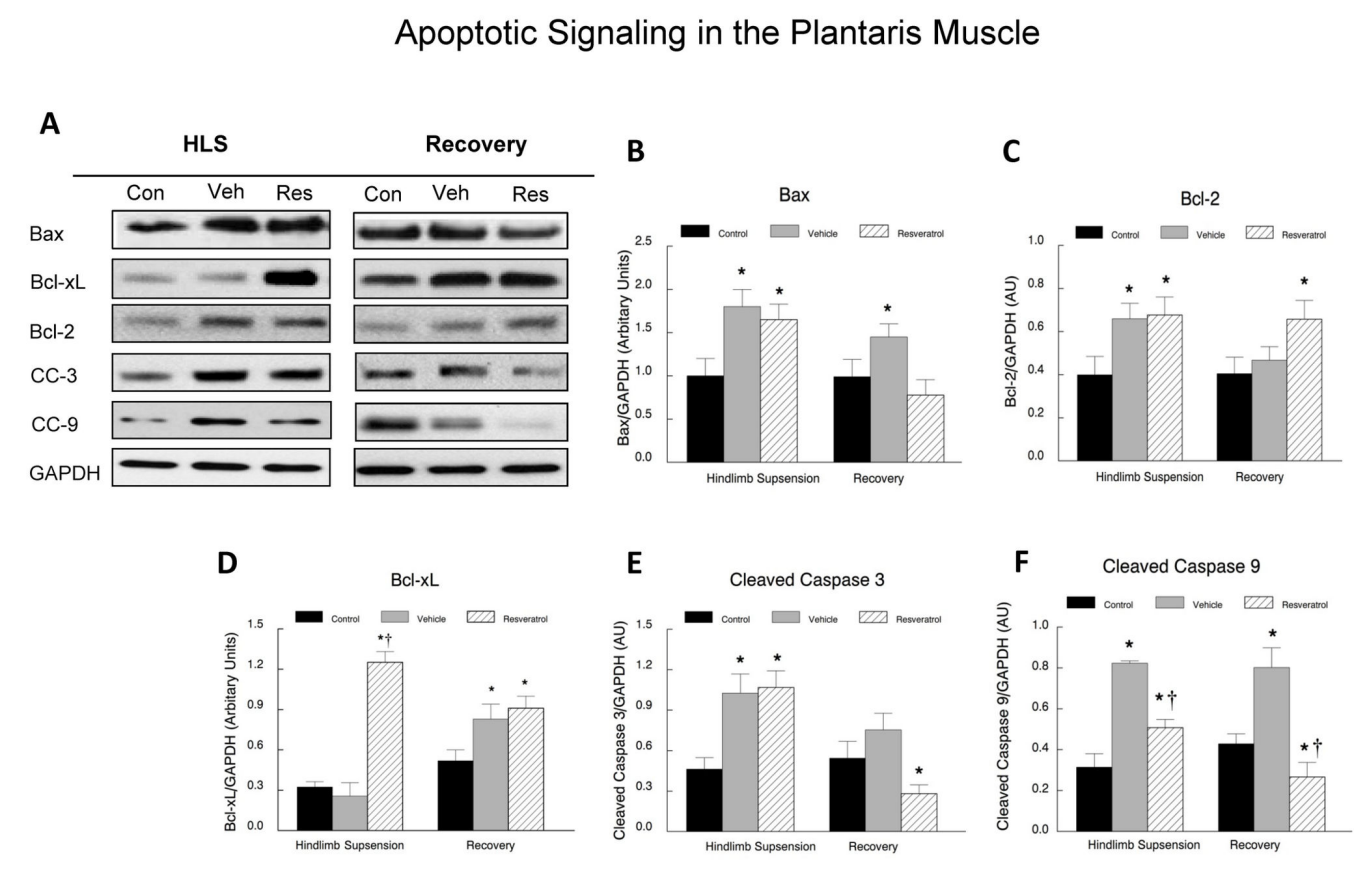
Apoptotic signaling proteins in plantaris muscles. Plantaris muscle samples were homogenized and total muscle lysates were separated on a 4-12% gradient polyacrylamide gel by routine SDS-PAGE. The proteins were electroblotted to nitrocellulose membranes and the signals were developed by chemiluminescence. **A**. Representative western blots of apoptotic proteins. Bax, Bcl-xL, Bcl-2, cleaved caspase 3 (CC-3), cleaved caspase 9 (CC-9) and GAPDH. The digital images were quantified as optical density x band area using ImageJ software and normalized to GAPDH, which was used as the loading control for each lane. The data are expressed as the protein signal to the GAPDH signal and are reported as mean ± SEM in arbitrary units. The western blots were run a minimum of three times for each protein, and the data were averaged for each animal’s data point. These data include: Bax (**B**) Bcl-2 (**C**), Bcl-xL (**D**), cleaved caspase 3 (**E**), and cleaved caspase 9 (**F**). * *P*<0.05 *vs*. cage control; † *P*<0.05 vehicle *vs*. resveratrol.

We have previously characterized apoptotic protein changes during hindlimb suspension and reloading [13,30]. In the current study, western blots were used to determine the relative content of anti-apoptotic Bcl-xL and Bcl-2 and pro-apoptotic Bax, cleaved caspase 3 and cleaved caspase 9 proteins, as representative of changes in the apoptotic signaling pathway. Protein levels of the respective proteins were normalized to GAPDH protein levels and were expressed in arbitrary units. Although resveratrol did not reduce the hindlimb suspension-induced elevation in Bax, in the recovery group, the pro-apoptotic Bax protein abundance was lower in resveratrol than vehicle treated plantaris muscles (Figure **10**). In a similar pattern, pro-apoptotic proteins cleaved caspase 3 and cleaved caspase 9 were elevated by hindlimb suspension, but the increases in cleaved caspase 9, a mitochondrial-associated pro-apoptotic protein was suppressed by resveratrol. Resveratrol suppressed both cleaved caspase 3 and cleaved caspase 9 in the plantaris muscles of the recovery group as compared to the vehicle treated group (Figure **10**). Bcl-2 was elevated in a similar fashion in vehicle treated and resveratrol treated plantaris muscles during hindlimb suspension (Figure 10). During recovery, the Bcl-2 protein abundance returned to control levels in the vehicle-treated plantaris muscle, but it remained elevated in plantaris muscles that were treated with resveratrol. The protein abundance of the anti-apoptotic Bcl-xL was significantly increased in the resveratrol group following hindlimb suspension, while no changes were observed in the vehicle (0.20 ± 0.16) treated group as compared to the cage control (0.28 ± 0.11) animals (Figure **10**). During recovery, the abundance of Bcl-xL was elevated in both the vehicle (0.83 ± 0.11) and resveratrol (0.91 ± 0.09) treated animals, although there were no differences observed between these two groups (Figure **10**).

**Figure 11:**
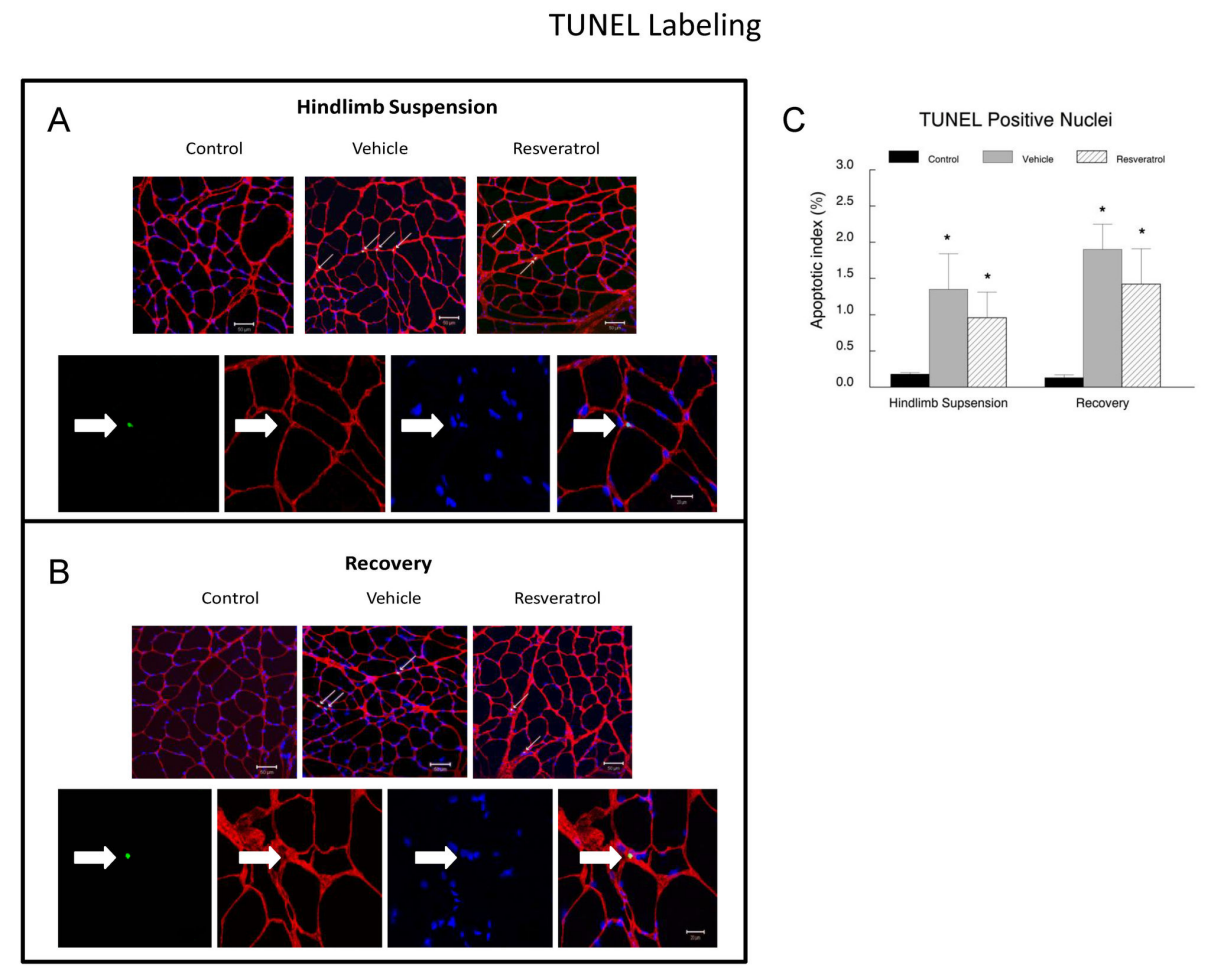
Immunocytochemical evidence for apoptotic nuclei in reloaded muscles. DNA fragmentation was assessed with TUNEL staining (green) as an indication of nuclei committed to apoptosis in the plantaris muscles from animals. The basal lamina of the muscle fibers was incubated in an anti-lamina antibody (red) to identify nuclei adjacent to or inside the basal lamina of the fibers. Representative tissue cross sections are shown for animals in the hindlimb suspension (**A**) and recovery (**B**) groups. **C**. Quantification of the TUNEL positive nuclei that were located at or below the basal lamina of the muscle fibers in the recovery or cage control animals. *P<0.05 vs. cage control.

To complement the western blot analysis, we used a TUNEL assay to confirm the relative amount of myonuclei undergoing apoptosis immediately following hindlimb suspension (Figure **11A**) and recovery (Figure **11B**). Consistent with our previous observations [13,30], there was a significant increase in apoptosis following hindlimb suspension (Figure **11C**). While insignificant, the mean increase in the apoptotic index was 29% higher in the vehicle (1.35 ± 0.49) treated animals compared to the resveratrol (0.96 ± 0.50) treated animals. In the recovery group, the TUNEL index remained significantly elevated in both the vehicle (1.90 ± 0.50) and resveratrol (1.42 ± 0.90) treated animals.

## Discussion

Recovery of skeletal muscle mass after immobilization-induced atrophy is difficult or impossible in aged animals or humans [8–10], but it is more complete in younger hosts. Therefore, the focus of our study was to examine the efficacy of resveratrol to improve muscle recovery following disuse in aging, and therefore we were not interested in identifying differences in response between young and old animals. In this study, resveratrol treatment failed to prevent the loss of plantaris muscle mass in old rats during 14 days of hindlimb suspension. However, resveratrol treatment enhanced the recovery of plantaris muscle mass during reloading after the period of disuse. The mechanism for resveratrol’s effect on muscle recovery after disuse appeared to be at least partially through a suppression of pro-apoptotic proteins and an increase in the abundance of anti-apoptotic proteins in reloaded muscles of resveratrol-treated animals as compared to vehicle-treated animals. Thus, it is possible that the lower apoptotic environment may have contributed to more total surviving myonuclei (by having a small increase in proliferation and less death of the new nuclei) and therefore provided a greater potential for nuclear regulation of muscle repair.

### Muscle and muscle fiber responses to resveratrol during reloading

As expected [13,19,31–33], a fourteen day period of hindlimb suspension induced significant reductions in plantaris muscle weights of the aged animals; however, resveratrol did not prevent or slow muscle wasting in response to disuse in the old rats that were examined in this study. This was an unexpected finding because a high dose (400 mg/kg) of resveratrol has been shown to protect soleus muscles from hindlimb suspension-induced atrophy [18], and we had previously found a strong trend (p=0.06) for a lower dose of resveratrol (12.5 mg/kg) to reduce disuse atrophy in the rat gastrocnemius muscles from old rats [19]. Furthermore, we increased the intake levels of resveratrol from our earlier study to 125 mg/kg/day in this study, expecting that the greater level would increase the potential for improving muscle recovery after unloading. While resveratrol and its metabolites were clearly present in plasma of treated animals that were examined in the current study (Table S1), and AMPK, pAMPK and PGC1α were all greater during the recovery period in the plantaris muscles of animals fed resveratrol as compared to vehicle fed animals (Figure 5), this dose of resveratrol may still not have been high enough to provide any significant sparing of muscle mass during hindlimb suspension. Nevertheless, these metabolic changes appeared to be adequate to provide modest but significant improvements in muscle mass and muscle fiber recovery as compared to vehicle-treated animals after resuming ambulation. Thus, it is likely that the protective effects of resveratrol against disuse atrophy and/or muscle growth to recover from disuse are muscle and/or dose specific. This may indeed be the case because resveratrol was unable to attenuate the hindlimb suspension-induced decreases during hindlimb suspension or improve muscle mass recovery in the soleus of these animals (data not shown). 

In contrast to hindlimb suspension, resveratrol treatment enhanced the recovery of plantaris muscle mass during reloading after the period of disuse. This appeared to be the result, at least in part, of a resveratrol mediated improvement in type IIA and type IIB fiber size as compared to the vehicle treatment. It is interesting to note that type I fibers were unable to regain the hindlimb suspension-induced lost muscle fiber size even after 14 days of reloading in either resveratrol or vehicle treated old animals (data not shown).

It was not surprising that resveratrol improved muscle fiber size, given that similar results have been observed in several recent studies [17,18]. These beneficial effects observed in our current study are likely due in part to the ability of resveratrol to attenuate muscle protein degradation during conditions which normally would induce atrophy [17] or stressful conditions that are resistant to hypertrophy. Although not significant, resveratrol tended to enhance the proliferation of muscle progenitor stem cells (e.g., satellite cells) during the recovery period, and once the cells had differentiated, they would be expected to provide a greater population of nuclei to participate in muscle re-growth. Thus, we cannot fully rule out the possibility that resveratrol increased the growth response during recovery following our suspension-induced atrophy protocol.

The rightward shift in the force-frequency curve especially during recovery after hindlimb suspension is suggestive of a faster muscle phenotype, which is consistent with our observations of a type I to type II fiber composition change in the plantaris muscles. While the proportion of fibers that expressed type I myosin heavy chain decreased in the plantaris from both the resveratrol and vehicle treated animals following suspension, there was a significant increase in the percentage of type IIB fibers during this period. The percent of type IIA and IIB fibers in the plantaris was not different between resveratrol and vehicle treated animals in the recovery period. This suggests that resveratrol has a minor or no effect on myosin expression under the hindlimb suspension and reloading conditions of this study, yet, it did improve the amount of protein in plantaris in a fiber type specific fashion in the reloaded muscles. This was shown by larger type IIA and IIB fibers in resveratrol treated animals of the recovery group. These results were slightly different than that reported in a study by Momken and colleagues [18]. Under similar experimental conditions as our present study, they observed that resveratrol supplementation attenuated the decrease in the proportion of type I fibers following suspension [18]. They attributed this finding to the ability of resveratrol, acting primarily through Sirt1, to increase the expression of PGC1α, which has been shown to promote the maintenance of slow-twitch fiber’s oxidative characteristics [34]. However, in our study, PGC1α protein levels increased but Sirt1 activity and protein abundance were not significantly improved in the resveratrol treated animals that were in the recovery group. Nevertheless, we do not know if the increase in PGC1α occurred in both type I and type II fibers of the fast glycolytic plantaris muscle that was examined in our current study, whereas the slow and more oxidative soleus muscles soleus was examined by Momken et al. [18]. The effects of resveratrol might be muscle specific because type I fibers in the plantaris of resveratrol and vehicle treated animals had similar reductions in cross sectional area after hindlimb suspension or recovery. Our data suggest that type II fibers may be sensitive to resveratrol-regulated recovery, whereas type I fibers are resistant to re-growth after a period of disuse at least at the doses of resveratrol and the ages of animals that were examined in this study.

### Resveratrol reduces apoptotic signaling during reloading

Resveratrol has been shown to provide beneficial effects on apoptosis in multiple different tissues [19,35–37]. Therefore, we examined if resveratrol altered the apoptotic signaling in the plantaris muscles of old rats during recovery from hindlimb suspension. This is important because apoptosis is elevated in skeletal muscle with aging and disuse [38–41]. As expected, hindlimb suspension increased pro-apoptotic signaling as evidenced by elevations in Bax, cleaved caspase 3 and cleaved caspase 9 in the plantaris muscles after hindlimb suspension. During the recovery period, resveratrol suppressed each of these pro-apoptotic proteins as compared to their protein abundance in vehicle treated animals. While resveratrol returned Bax to control levels in the plantaris muscles of the recovery animals, cleaved caspase 3 and cleaved caspase 9 protein abundance fell below control levels during the recovery period in the plantaris muscles of resveratrol treated animals. This resveratrol-enhanced reduction in pro-apoptotic signaling was accompanied by an improvement in Bcl-2 protein abundance during recovery, although Bcl-xL was not different in vehicle and resveratrol treated animals of the recovery group. While TUNEL index values were slightly lower in the resveratrol treated animals of the recovery group as compared to the vehicle treated animals, this did not reach statistical significance. Nevertheless, this small change may have been physiologically relevant because we do not know how many nuclei need to be spared from dying in order to improve muscle hypertrophy. Coupled with the increases in Bcl-2 and the reductions in Bax, cleaved caspase 3 and cleaved caspase 9, it is possible that these changes to a muscle environment that is less favorable for apoptosis, could have prevented the loss of some nuclei, which may have contributed to an improvement in muscle recovery, especially in type II fibers, after disuse in the animals of this study. It should be noted that other compounds have also been shown to reduce apoptotic signaling in this model of unloading and loading, including HMB [13] and therefore, apoptotic signaling might be a good target to reduce muscle wasting and/or improve muscle recovery after disuse in aging.

### Muscle function

The fatigue index revealed a decrease in the ability of the plantaris to withstand repeated contractions in both of the groups subjected to suspension or after reloading. It was unexpected that after reloading following hindlimb suspension, the resveratrol treated animals would have a reduced fatigue index as compared to the control group while the vehicle treated animals had a similar fatigue index as the control animals. The lower fatigue index in the resveratrol treated animals occurred, despite having greater PGC1α levels which would have been expected to stimulate mitochondrial biogenesis. Nevertheless, the larger type IIA and type IIB fibers in the resveratrol treated muscles of the recovery group would not be expected to confer improved fatigue resistance, so it is possible that the larger mitochondrial poor fibers in the plantaris might have diluted the anticipated improvement in fatigue resistance by resveratrol. However, the observation that resveratrol treatment does not improve upon the fatigue index is not without precedent. In other studies involving resveratrol supplementation [26,42], no improvements were observed in muscle fatigability. Nonetheless, it must also be noted that in one study in which young, dystrophic mdx mice were supplemented with resveratrol, there was a significant improvement of the fatigue index [43]. It is possible that the effect of resveratrol on muscle fatigue might be specific to the experimental models, dose of resveratrol, and intervention that was employed. Therefore, further research in the effects of resveratrol on muscle fatigue is needed to reconcile these discrepancies. 

### Study limitations

The potential limitation of this study is that the dose of resveratrol used, while higher than another study which showed reduced oxidative stress [44] was simply not enough to elicit the full benefits of its regenerative capabilities on all fiber types. It is possible that reduced absorption in the gastrointestinal track of the old rats, or increased rates of metabolism of resveratrol as a first past through the liver, may have reduced the effectiveness of the compound during the high stress conditions of unloading and reloading. Furthermore we do not know why the plasma levels of resveratrol decreased in the recovery animals as compared to the hindlimb suspension animals. If this also occurred during long-term resveratrol use (perhaps by elevating the liver associated metabolism of resveratrol), this might explain why resveratrol, which reduced muscle levels of oxidative stress, was unable to attenuate muscle losses due to aging, when it was given to mice over a period of 10 months [26]. While, the resveratrol administered by gavage in the current study, was the same in both groups, we cannot rule out the possibility that the dietary intake of resveratrol in the food differed under hindlimb suspension and recovery conditions. Nevertheless, our observations in other studies using the suspension and reloading model, have not detected a reduction in food intake in the recovery vs. the unloading period [30]. Thus, we speculate that the most likely explanation is that resveratrol metabolism might have differed (increased) during the recovery period as compared to the hindlimb suspension condition. Nevertheless, the evidence from this study provides a basis for conducting further research to optimize the dose or timing of resveratrol supplementation in aging and perhaps examine resveratrol metabolites in more detail during unloading and reloading. In addition, translational research is needed to evaluate resveratrol’s potential as a countermeasure to reduce the decline of muscle function or mass in the elderly following periods of forced disuse. 

### Summary

The findings from our present study provide evidence for the modest but potentially important benefits of resveratrol supplementation in improving skeletal muscle regeneration following a period of disuse with a subsequent recovery in aging. Although resveratrol did not prevent either body or muscle weight loss following hindlimb suspension, it was able to induce favorable changes to type IIA and type IIB muscle fiber CSA and reduce apoptotic signaling in muscles of old animals. This is particularly relevant in the elderly population which experience preferential atrophy of type II fibers in sarcopenia. 

## Supporting Information

Table S1 **Plasma levels of resveratrol and its metabolites were determined in triplicate from 100 µl of plasma.** The data are recorded as ng/mL of plasma and are reported as mean ± SEM (n=12 animals/group). Although no resveratrol was detected in vehicle treated animals, traces of its metabolites were present in the plasma, suggesting that the diet of vehicle treated animals had very low (but detectable levels of metabolites (although some vehicle treated animals had no detectable levels of any resveratrol metabolite).
(DOCX)
