# Supplementary material for: Effects of Resveratrol on the Recovery of Muscle Mass Following Disuse in the Plantaris Muscle of Aged Rats
Source: PLoS One. 2013 Dec 12;8(12):e83518. doi: 10.1371/journal.pone.0083518 (PMC3861503; doi:10.1371/journal.pone.0083518)
Supplement: Table S1 — Plasma levels of resveratrol and its metabolites were determined in triplicate from 100 µl of plasma. The data are recorded as ng/mL of plasma and are reported as mean ± SEM (n=12 animals/group). Although no resveratrol was detected in vehicle treated animals, traces of its metabolites were present in the plasma, suggesting that the diet of vehicle treated animals had very low (but detectable levels of metabolites (although some vehicle treated animals had no detectable levels of any resveratrol metabolite). (DOCX) [file pone.0083518.s001.docx]

**Table S1:**

**Plasma levels of Resveratrol, Resveratrol Sulfate, 3-Glucuronide and 4-Glucuronide**

| **Plasma levels (ng/mL)** | | | |  |
| --- | --- | --- | --- | --- |
| **Resveratrol** | **Resveratrol Sulfate** | **3-Glucuronide** | **4-Glucuronide** |  |
| **Vehicle-Fed** | 0 ± 0 | 0.441 ± 0.04 | 4.2 ± 0.70 | 1.70 ± 0.42 |
| **Resveratrol- Treated** | 400.3 ± 69.9 | 331.3 ± 56.9 | 1865.9 ± 406.2 | 1914.6 ± 449.0 |
